# Botulinum Toxin Injection to Treat Bruxism in a Child: A Case Report and Technique Description

**DOI:** 10.7759/cureus.103108

**Published:** 2026-02-06

**Authors:** Franco Ignáccio Mallaguti, José Geraldo Malaguti, Otacilio de Paula Rodrigues Júnior

**Affiliations:** 1 Implantology, Instituto Malaguti, Uberaba, BRA; 2 Periodontology, Instituto Malaguti, Uberaba, BRA

**Keywords:** botulinum toxin, bruxism, orofacial pain, pediatric dentistry, temporomandibular disorders

## Abstract

Managing sleep bruxism (SB) in children remains a clinical challenge due to the limited evidence-based treatment options for muscle hyperactivity in this population. This case report describes the successful use of botulinum toxin type A (BTX-A) in managing SB in an otherwise healthy eight-year-old girl. The patient received 10 units of BTX-A in each masseter muscle, administered at two points per side using anatomical landmarks and muscle palpation to guide injection. Symptom resolution was reported as early as the day after treatment, with effects lasting approximately two months and no adverse reactions observed. This is the first known report of BTX-A being used to treat bruxism in a healthy pediatric patient. The findings suggest that botulinum toxin may be a safe and effective alternative for managing muscle hyperactivity in children. Further research is needed to establish optimal dosing protocols and long-term safety.

## Introduction

Originally, bruxism was defined as a repetitive masticatory muscle activity characterized by clenching or grinding of the teeth and/or by bracing or thrusting of the mandible and specified as either sleep bruxism (SB) or awake bruxism (AB) [1]. However, four years later, this definition had to be revised for some points to be clarified. As a result, in 2017, the international consensus on the assessment of bruxism suggested two new, distinct definitions as separated entities for SB and AB [2].

Irrespective of being SB or AB, studies have been focusing almost solely on the adult population, and pediatric scientific data are scarce [3]. There is a high range of variability in pediatric bruxism prevalence of about 3.5% to 40.6%, assessed in systematic reviews [4]. Nevertheless, it is of utmost importance to know how to diagnose and treat children with bruxism, since they may experience a higher frequency of joint clicks, muscle fatigue, difficulties in yawning, headaches [5], masticatory muscle tenderness, periodontal problems, tooth surface loss and hypersensitivity [6], and are almost three times more likely to develop temporomandibular disorders than non-bruxer children [7].

Bruxism should be accurately diagnosed using instrumental assessments, like polysomnography, and non-instrumental assessments, like self-report and clinical inspection [2, 3]. However, the instrumental method may not work well in children because it requires more collaboration from them, in addition to the difficulties of using a sleep laboratory [3]. Therefore, due to these limitations, the non-instrumental method ends up being more suitable for children, with the parents' report to the clinicians being of fundamental importance, always accompanied by the appropriate clinical examination [3]. Interestingly, Cheifetz et al. [8] demonstrated that parents who keep their bedroom door open indicate a 1.7 times greater incidence of SB in their children than those who keep it closed. 

Managing bruxism in children is a challenge. Lots of methods were proposed to treat it, from simply following its evolution [3] to homeopathic remedies, photobiomodulation, hydroxyzine [9], and occlusal appliances [3]. The first two cited approaches have low to no toxicity and are very promising [9]. Hydroxyzine was suggested to be prescribed only in severe cases due to its mild side effects, like fatigue [9]. And occlusal appliances might restrict maxillary growth in children [3].

Since both aforementioned bruxism definitions start with "masticatory muscle activity”, it is clear that the main treatment goal should be regulating muscle activity. Although the phenomenon is coordinated by central neuromodulatory pathways [2], its clinical expression is fundamentally a peripheral muscular event [1]. The use of botulinum toxin is arising as a really promising option to control bruxism in adult patients, also relieving pain in their jaw muscles [10], and requires no patient compliance. This endotoxin is produced by a Gram-positive anaerobic bacterium called *Clostridium botulinum*, and when injected into human muscles in therapeutic doses, botulinum toxin A (BTX-A) cleaves SNAP-25 in the cellular cytosol, preventing neurotransmitter release and thus diminishing muscle activity [11]. 

To date, there are few reports of botulinum toxin injection to manage bruxism in pediatric patients with brain injury [12], autism [13], or Rett’s syndrome [14], but no reports on healthy children. Therefore, based on the past cited success reports, this paper aims to describe a successful case of pediatric bruxism management with botulinum toxin injection.

## Case presentation

The patient, an eight-year-old female, presented to our clinic accompanied by her mother, who complained about listening to her daughter grinding her teeth while sleeping every night and often waking up with the noise. It started when she was younger, stopped during orthodontic treatment, and resumed recently, when the treatment ended. Her mother also reported that the former orthodontist manufactured a retainer to keep the patient's dental arches apart, but the patient did not get used to it. They also tried consulting with other pediatric dentists, who did not manage to control the patient's teeth grinding. At the clinical examination, generalized dental wear was observed, with prominent facets on the canines and deciduous molars. Although the patient did not report spontaneous orofacial pain, bilateral tension and volume were noted upon palpation of the masseter muscles, and the mother described the nightly grinding noise as 'constant and loud enough to be heard from an adjacent room,' indicating high intensity. Therefore, the authors graded the patient as Grade 2, that is, probable SB, based on a positive clinical exam and a positive report from her mother [2], and thus, suggested botulinum toxin injection into the patient’s masseter muscles. The present case report was prepared following the CARE guidelines to ensure transparency and accuracy in the reporting of clinical findings and treatment outcomes [15].

The patient presented with an unremarkable medical history, with no prescribed medications, systemic or psychological disorders, or known allergies.

**Figure 1:**
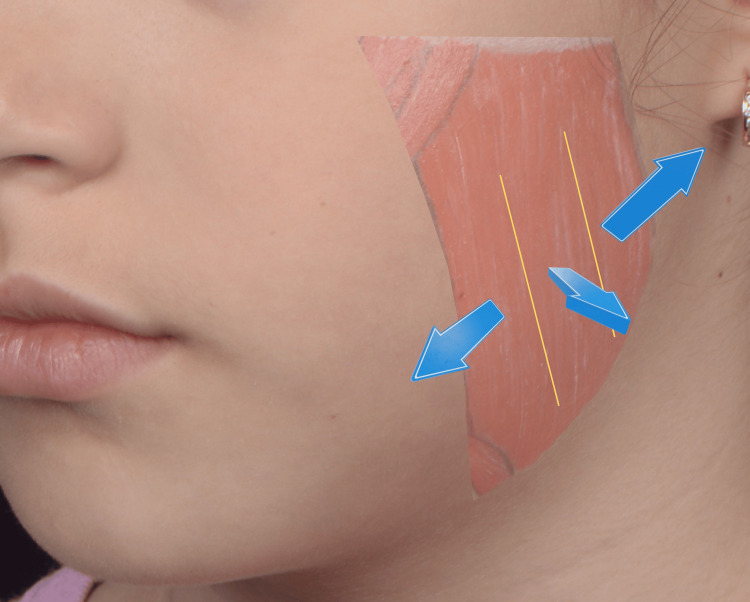
Anatomical landmarks for masseter muscle injection determined by palpation during voluntary clenching The injection points are determined through manual palpation during voluntary clenching. Posterior displacement of the clinician’s finger identifies the posterior part of the masseter, while outward displacement identifies the central region. The anterior portion of the masseter is intentionally avoided to prevent diffusion to the risorius muscle, thereby minimizing the risk of functional and aesthetic adverse effects, such as smile asymmetry. Note: The parents of the patient consented to have the patient's images used in an open-access publication. A written and signed consent statement was provided to the journal.

**Figure 2:**
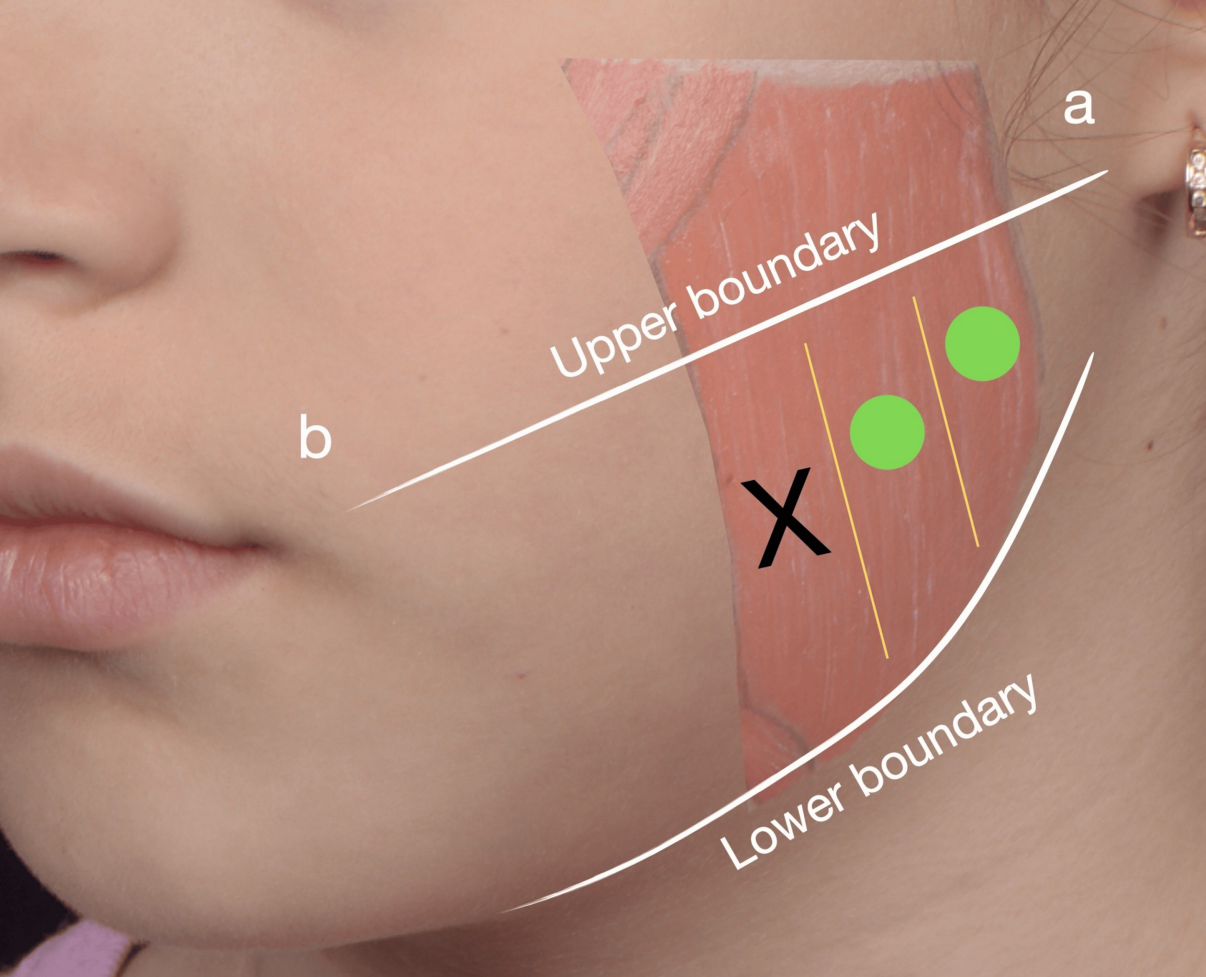
Injection points diagram for masseter muscle treatment with botulinum toxin type A The upper boundary is defined by a line drawn from the earlobe (a) to the oral commissure (b), while the mandibular edge defines the lower boundary. Within these limits, injection sites are determined by palpation during voluntary clenching to identify the muscle's three portions. Green circles indicate the posterior and central regions of the masseter, representing safe and effective injection points. The black 'X' denotes the anterior masseter, an area intentionally avoided to prevent toxin diffusion to the risorius muscle, thereby minimizing the risk of adverse functional or aesthetic effects, such as smile asymmetry. Note: The parents of the patient consented to have the patient's images used in an open-access publication. A written and signed consent statement was provided to the journal.

Hence, the authors decided to inject each masseter with 10U of BTX-A (Botox, Allergan Aesthetics, Irvine, CA, USA) reconstituted with 0.5 ml of saline, divided into two points (posterior and central parts) of 5U, using a 30U insulin syringe coupled with a 30G x 8 mm length needle (BD Ultrafine II, Becton, Dickinson and Company, Franklin Lakes, NJ, USA). Needle length was chosen according to children’s hypertrophic masseter thickness [6]. The injection points were determined using the following anatomical landmarks: (1) a line from the earlobe to the labial commissure was used to define the upper boundary; (2) the jawline served as the lower boundary [16]; (3) within these limits, the injection sites were identified based on muscle contraction. The clinician gently places a finger on the skin over the masseter muscle while the patient is asked to clench their teeth. If the muscle contraction passively displaces the clinician’s finger posteriorly, the finger is positioned over the posterior region of the masseter, and an injection point should be placed at the area of maximal muscle strength. If the finger is displaced directly outward toward the clinician, it indicates the central region of the masseter, where another injection point should also correspond to the area of strongest contraction. However, if the finger is pushed forward, in the direction of the patient’s mouth, this suggests contact with the anterior region of the masseter. In this case, injection of BTX-A is not recommended, as this area is close to the risorius muscle, increasing the risk of adverse effects such as smile asymmetry or compromised smile function (Figures 1-2).

Thirty minutes before the injection, 4% lidocaine numbing cream (Dermomax, Biosintética, São Paulo, SP, Brazil) was applied on the skin above the masseter regions, then the injection sites were marked following the previous description. Post-injection instructions were not to touch the injection sites, apply ice onto them, or lie down, and to avoid sun exposure, vibrations, and physical exercise [17,18] for four hours to limit diffusion and migration of BTX-A to unintended sites [12].

On the day following the BTX-A injection, the patient’s mother reported a complete absence of bruxism-related signs: no grinding noises were heard, and the patient experienced uninterrupted sleep throughout the night, without waking herself or others. At the 15-day postoperative follow-up appointment, a comprehensive assessment was conducted, including a patient interview regarding post-injection experiences, standardized photographs to document any aesthetic changes, and a clinical examination. During palpation, a notable reduction in masseter muscle tension and contraction force was observed, confirming muscle relaxation. No adverse effects were reported by the patient or observed clinically. Continued follow-up was maintained via text messaging with the patient’s mother. According to her reports, the patient remained free of teeth grinding and associated noises for approximately two months post injection. Thereafter, symptoms gradually reemerged, returning to baseline frequency and intensity by the end of the third month. Given the rapid return of symptoms and the high muscle tone observed previously, the clinical team elected to increase the dosage in the subsequent injection in an effort to prolong the duration of therapeutic benefits. The chronological progression of the case, including the diagnosis, intervention, and follow-up milestones, is summarized in Table 1 [12,15,19].

**Table 1:** Chronological timeline of clinical presentation, intervention, and follow-up according to CARE guidelines. Reference: [15]

Date/Period	Clinical Event/Intervention
Day 0	Initial consultation, clinical exam, and diagnosis of probable sleep bruxism (Grade 2).
Day 0 (Procedure)	Injection of 10U of botulinum toxin type A in each masseter muscle.
Day 1	Immediate cessation of teeth grinding sounds was reported by the mother.
Day 15	Postoperative follow-up: clinical exam confirmed muscle relaxation; no adverse effects.
End of Month 2	Maintenance of therapeutic effect; initiation of gradual reemergence of grinding sounds.
End of Month 3	Return of symptoms to baseline intensity; clinical team evaluates dosage adjustment.

## Discussion

Pediatric bruxism remains significantly less studied than its adult counterpart, and current scientific data on its epidemiology are inconclusive. One study reported a wide prevalence range of 3.5% to 40.6% [4], highlighting the variability in diagnostic criteria and methodologies. A more recent investigation found a 28.3% prevalence rate among children aged two to six years [20]. Similarly, Drumond et al. reported a 40.0% prevalence of probable SB in children aged eight to 10 years, with higher rates observed in males and in those with a history of nail- or object-biting behaviors considered potential mechanisms for tension release [21].

The consequences of pediatric bruxism can be significant and should not be overlooked, as affected children are reportedly three times more likely to develop temporomandibular disorders compared to non-bruxing peers [7]. Additionally, 98.09% of children between two and six years of age with bruxism reported experiencing dental pain [20]. Other commonly associated symptoms include restricted mouth opening [9], increased frequency of headaches, muscle fatigue, joint clicking, and difficulty yawning [5]. Furthermore, clinical manifestations may involve masticatory muscle tenderness, periodontal issues, tooth surface loss, and dentin hypersensitivity [6].

A variety of treatment approaches for pediatric bruxism have been explored in the literature. Among them, photobiomodulation has emerged as a particularly promising option due to its non-invasive, painless nature and lack of toxicity. However, it typically requires multiple sessions to achieve symptom relief, necessitating consistent cooperation from both the child and their caregivers [9].

Pharmacological intervention with hydroxyzine has also demonstrated efficacy; however, its long-term safety remains uncertain, and it may cause mild side effects. As such, its use is generally reserved for more severe cases [9].

Homeopathic remedies, including *Melissa officinalis* and *Phytolacca decandra*, have been suggested as alternative treatments due to their low cost, high accessibility, minimal toxicity, limited side effects, and lower dependence on patient compliance. Nonetheless, their effectiveness is inferior when compared to other modalities [9]. Similarly, aromatherapy has been proposed for managing pediatric bruxism, though current evidence supporting its efficacy is rated as low to very low [22].

In adults, the most commonly recommended intervention is the use of an oral appliance or occlusal splint [23], due to its conservative nature and cost-effectiveness [10]. However, such devices may not be suitable for pediatric patients, as they typically demonstrate lower adherence and may risk interfering with maxillary growth and development [3].

The use of botulinum toxin to manage masticatory muscle parafunction in adults is a well-established clinical procedure, supported by robust scientific evidence. It has been shown to be more effective than other commonly recommended treatments, such as oral splints [10]. Regarding pediatric use, the safety of botulinum toxin has been demonstrated in several studies involving children with neurological and developmental conditions, including those with brain injuries [12], autism spectrum disorder [13], and Rett syndrome [14].

However, to our knowledge, this is the first reported case of botulinum toxin being used to manage masticatory muscle hyperactivity in a systemically healthy pediatric patient. Consequently, the targeted muscles and administered doses in this case were primarily guided by the limited literature available on its use in children with systemic conditions. For instance, Pidcock et al. [12] and Monroy and Fonseca [13] injected their patients’s masseter muscles with a total of 15 units per side. In contrast, Komisarek et al. [14] employed a higher dosage, totaling 100 U of botulinum toxin distributed at their patient’s masseter and temporalis muscles, based on a dosing protocol adapted from a pilot study in adult patients.

Moreover, the authors' clinical expertise and commitment to professional ethics were fundamental in formulating the initial treatment protocol. Given that the patient was a healthy child presenting solely with nocturnal teeth grinding, without associated pain, functional impairment, or other symptoms, a conservative approach was adopted. An initial dose of 10 units per masseter was deemed appropriate for the first application. The patient’s mother was instructed to provide regular feedback regarding the treatment’s outcomes, allowing for dose adjustments in future sessions if necessary, with the goal of optimizing both therapeutic efficacy and patient comfort.

In adults, the standard target muscles for managing bruxism symptoms are the masseter and temporalis muscles [23]. However, in the present case, the temporalis muscles were intentionally excluded from the injection protocol to minimize potential risks of impairing chewing and swallowing functions. This decision may have influenced the overall duration of the therapeutic effect, as existing evidence suggests that both the efficacy and longevity of botulinum toxin are dose-dependent [19]. Nonetheless, a study by da Silva Ramalho et al. demonstrated that protocols targeting either the masseter alone or both the masseter and temporalis muscles are equally effective for up to 120 days in adult patients [24].

In the present case, clinical improvement was observed as early as the day after injection and lasted for about two months, with symptoms gradually returning after three months. In contrast, Pidcock et al. reported marked improvement at four days, moderate at 30 days, and minimal at two months [12]. Despite similar injection sites, volume, and diluent, and a higher dose in their study, two factors may explain our patient's quicker and longer response: Pidcock’s patient likely had more severe bruxism, and their use of ice post injection may have delayed onset and reduced efficacy [18]. Similar observations apply to the study by Monroy and Fonseca [13], who also reported onset and duration timelines comparable to those of Pidcock et al. [12]. However, their report lacked detail regarding injection point localization, reconstitution technique, solution used, and whether cryotherapy was applied, making direct comparisons challenging.

The development of immunogenicity is one of the primary concerns associated with repeated administration of BTX-A. Circumstantial evidence suggests a potential link between the formation of neutralizing antibodies (NABs) and the presence of non-toxic accessory proteins in certain BTX-A formulations [18]. Additionally, high cumulative doses and short intervals between injections are recognized risk factors for NAB development [18]. This risk may be even more pronounced in pediatric populations, who are reported to have a higher prevalence of NABs compared to adults, likely due to the relatively higher dose per kilogram of body weight administered in children [25]. In light of these considerations, current best practices recommend using the lowest effective dose and maximizing the interval between injections [18]. According to the manufacturer's guidelines, Botox should not be administered more frequently than every three months [26]. Accordingly, the present authors opted to wait four months before administering a second, higher dose to our patient, in contrast to the protocol followed by Pidcock et al. [12], who performed a second injection just two months after the initial one.

We recognize certain limitations in our observations. This case report relies primarily on qualitative observations and parental reports. While these provide valuable clinical insights into the patient’s daily experience and the treatment's impact, the absence of objective measurements such as polysomnography or electromyography limits the quantitative assessment of muscle activity. Additionally, the follow-up for safety was conducted through clinical inspection and interviews rather than standardized safety scales. Future studies with larger pediatric cohorts and objective monitoring are necessary to establish standardized protocols for botulinum toxin use in healthy children.

## Conclusions

The use of BTX-A in this pediatric case demonstrated a temporary cessation of SB symptoms and provided immediate clinical relief for the patient. However, as this is a single-case report based on qualitative clinical observations, these findings should be considered preliminary. While BTX-A appears to be a feasible therapeutic option for managing muscular hyperactivity in children when traditional treatments fail, further controlled clinical trials with objective monitoring and long-term follow-up are essential to establish its definitive safety and efficacy in the pediatric population.
